# Complex Impedance Analyses of Li doped ZnO Electrolyte Materials

**DOI:** 10.1038/s41598-020-65075-0

**Published:** 2020-05-19

**Authors:** Shalima Shawuti, Atta ur Rehman Sherwani, Musa Mutlu Can, Mehmet Ali Gülgün

**Affiliations:** 1 Faculty of Engineering and Natural Science, Sabancı University, Tuzla Istanbul, Turkey; 2 Renewable Energy and Oxide Hybrid Systems Laboratory, Department of Physics, Faculty of Science, Istanbul University, Vezneciler Istanbul, Turkey

**Keywords:** Energy science and technology, Fuel cells

## Abstract

The recent studies indicate that internal point defects in solid electrolytes modify the electronic and ionic conductivity and relaxation mechanism of solid oxide fuel cells. We focused on synthesis of Lithium (Li) doped Zn_1-x_Co_x_O (x = 0.00, and 0.10) nanoparticles employing chemical synthesis technique with a reflux setup under constant Argon gas flow. The structural characterizations were performed by x-ray powder diffractometer (XRD) and x-ray photoelectron spectroscopy (XPS). Then, Rietveld refinements were performed to investigate the replacement of Li atom amount in ZnO lattice. Moreover, the variations in ionic conduction dependent on 5, 10 and 20 mol% Li doped ZnO were analysed via ac impedance spectroscopy. The complex measurements were performed in an intermediate temperature range from 100 °C to 400 °C. Ac conductivity responses of each sample were disappeared at a certain temperature due to becoming electronic conductive oxides. However, this specific temperature was tuned to high temperature by Li doping amount in ZnO lattice. Furthermore, the activation energy change by Li dopant amount implied the tuneable ionic conduction mechanism.

## Introduction

Discovery of oxide semiconductors are sparked a new field of materials due to easy producibility, tuneable surface reactivities, electrophysical and easily coating on any surface^1,2^. Low cost in production and suitable physical properties to wide variety of technological applications make ZnO as an important alternative candidate material for scientific researches. Especially, for solid oxide fuel cells (SOFCs) because of low electrical conductivities even for high temperatures (>300 ^o^C) due to their wide band gaps. Low electrical conductivities and stability on wide range of environmental conditions make oxide semiconductors be suitable for sense and adsorption of fuel gases^3^. In addition, specific surface area and surface defect amount of oxide semiconductors can be also tuned. In recent studies the polaron conduction is widely investigated subject such as chalcogenide glassy systems depend on partial amorphous phase ratio in a crystal structure, defect amount in lattice, defect type and dopant atoms^4–6^.

Desired ionic conductivity at operating temperature with low electronic conductivity are the most important property of electrochemical devices, especially for solid oxide fuel cells (SOFCs)^7,8^. Moreover, ionic conductivity mechanism through the grain-boundaries is the other investigated subject for SOFCs. Thus, high specific surface area and controllable surface defect amount enhance the usage ability of oxide semiconductors as a SOFC with high efficiency. The effects of grain boundary size change were also investigated for oxide semiconductors in previous study^9^. As well as boundary size, the boundary environments were also investigated subject^10–13^. The ionic conductivity performance of oxide semiconductors as an electrolyte material in a Na_2_CO_3_ (Sodium Carbonate) matrix is widely interested subject in SOFCs^10^ to decrease the working temperature of fuel cells with high ionic conductivity performances. The other studies also show that p-n heterojunction composite electrolytes create low-temperature operation of solid oxide fuel cells with good enough ionic conductivity values^11–13^. For example, ZnO based composite electrolytes such as ZnO-LCP (La/Pr doped CeO_2_) and BaCo_0.4_Fe_0.4_Zr_0.1_Y_0.1_O_3-δ_-ZnO with ionic conductivity values of 0.156 Scm^−1^ (at 550 ^o^C) and 0.098 Scm^−1^ (at 500 °C), respectively^11–13^. Furthermore, increased in dopant atom amount in oxide semiconductors modified the ionic conductivity, operating temperature, relaxation mechanism and cycle performance in a solid oxide fuel cell^14,15^. Among the dopant atoms, lithium (Li) is the most promising elements for SOFCs^16,17^. As mentioned in literature, thermal stability, electrochemical stability and low leakage currents are the possible advantages of Li based solid oxide fuel cells^14^. Furthermore, for recent technological applications, the most important advantage of Li doped oxide semiconductors are being suitable electrolyte materials for low temperature solid oxide fuel cells^17–20^.

ZnO is a wide band gap semiconductor with naturally an n-type electronic carrier. On the other hand, doping ZnO with transition metals and alkali metals can create p type conductivity. Therefore, doping atoms modify electronic properties as well as the ionic conductivity of ZnO.

P-type conductivities were observed for Li doped ZnO semiconductors and high amount of Li doping (16 mol% doped ZnO) created electrically insulator samples^21–23^. Li doping amount in ZnO created internal point defects of Li substitutions and interstitials Li atoms, which modify the electronic and ionic conductivity and relaxation mechanism by temperature. In the literature, it is mentioned that Li atoms can be easily incorporated into ZnO lattice^21–23^ and Li atoms reduce Zn vacancies defect amounts in ZnO lattice^21^. Li doping created a high densification, which enhancing the ionic conductivity, in a material^19^. Furthermore, Zn vacancies modifies the p-type conductivity in ZnO lattaice and low amount of Zn vacancies enhances n type conductive of ZnO nanoparticles. The studies assigned the nanosize p-n heterojunctions in composite electrolytes create low-temperature operation of solid oxide fuel cells with high ionic conductivity values^11–13^. Thus, the low amount of Zn vacancies causes to form n type nanoparticles and on the other side Li doped ZnO partciles perform p type conductivity. The formation of p-n heterojunctions enhances the ionic transportability of oxide semiconductor^24^. In addition to all, the researchers also prove high ionic conductivities of Li^+^ ions, especially for glassy solid electrolytes^25–27^.

In this study, lithium (Li) doped Zinc Oxide (ZnO) particles were synthesized via chemical route and ionic conduction mechanism by Li amount in ZnO lattice were investigated for understanding suitability as electrolyte materials in SOFCs. Reitveld refinement were used to understand the Li atoms replacements in lattice. Furthermore, spectroscopic techniques such as x-ray photoelectron spectrometer and UV-Vis. spectrophotometer were employed to prove the calculations. The ionic conductivity, ionic transport activation energy and ionic transport relaxations mechanism by temperature were analyzed by fitting the Nyquist plots obtained via ac impedance spectrometer.

## Experimental

Zinc acetate dehydrate (Zn(CH_3_COO)_2_.2H_2_O), Cobalt(II) acetylacetonate (Co(C_5_H_7_O_2_)_2_ and Lithium carbonate (Li_2_Co_3_) (all chemicals bought from Aldrich) were used as precursors. According to the stoichiometric ratio, the required amount of starting materials were mixed with 1 M solvent of Citric acid (C_6_H_8_O_7_) in deionized water to prepare a solution.

The suspension was heated and optimized around 80 ^o^C for about 180 minutes in a round bottom flask in oil bath with water cooling system and stirred, continuously, with a magnetic stirrer bar. Argon (Ar) gas flow was let into the solution by a gas inlet, and then passed through the condenser into the oil bubbler to get rid of the contaminating gases. Finally, this solid wet phase was dried in an oven at 300 °C for 8 h.

Thermal analysis were performed by synthesized particles under dry nitrogen flow with 40 ml/min in a α-Al_2_O_3_ reference container to monitor chemical reactions employing DTG-60H (TGA/DTA) 6300 spectrometer (Shimadzu Corp.).

The structural analyses were performed employing a Bruker brand x-ray powder diffractometer (XRD) with Cu K_α_ radiation (1.5418 Å) and a x-ray photoelectron spectroscopy (XPS) spectrometer equipped with AlK_α_ monochromatic x-ray exciting radiation with a resolution ~ 0.1 eV. The surface analyses were performed via scanning electron microscopy (SEM) micrographs. Reitveld refinements were also performed on the XRD patterns by employing FullProof subprogram^28^. In order to perform Reitveld refinement, each XRD pattern was measured by from 10° to 90° with 0.008 °/min steps.

Ionic transportation in synthesized samples were obtained employing a two-probe AC impedance spectrometer, Solartron 1260 and 1286, with an electrochemical interface. Electrochemical Impedance spectra (EIS) measurements were performed from 0.01 Hz to 13 MHz frequency range and 100 mV AC bias voltage amplitude under air atmosphere in temperature range of room temperature and 600 ^o^C. Before measurement, each powder was pressed under 300 MPa pressure to create 10 mm in diameter and 1 mm thick pellets. Each side of pellet was covered with silver paste to create Ohmic contacts with platinum electrodes of electrochemical interface.

## Results

**Figure 1:**
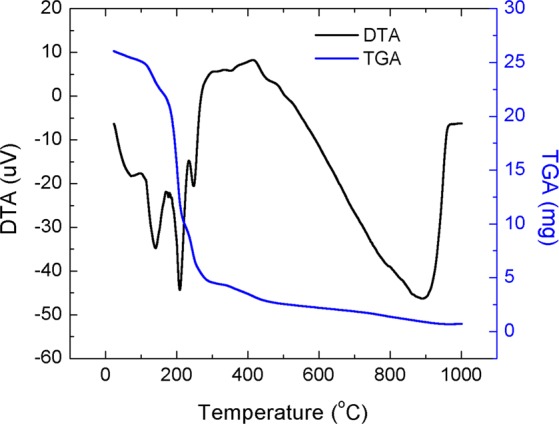
Differential and Thermo Gravimetric Analyzer (DT-TGA) spectra of prepared mixture, 10 mol% Li doped ZnO, by sol-gel tecnique.

Li atom amount depending ionic transport ability of ZnO semiconductors was targeted to synthesis via combination of chemical route and thermal treatment at high temperatures. After chemical route, a gel state mixture was obtained, and the temperature dependent chemical formations were analysed by a differential thermal and thermogravimetric spectrometer (DT-TGA). DT-TGA spectra of a mixture, prepared for 10 mol% Li doped ZnO, was demonstrated in Fig. 1. The spectra in figure was recorded from 30 to 1000 ^o^C, at which temperature no chemical reactions were observed.

The evaporations of possible organic chemicals were occurred by a mass loss with four different endothermic reactions in temperature ranges from 30 to 200 °C, from 290 to 315 °C and from 300 to 500 °C due to evaporation of water^29^, losing pure citric acid^30^, losing weakly bonded functional groups such as COOH on the surface^30^, and completing chemical reactions, respectively. The last endothermic reaction without mass loss was measured in temperature range of 500 and 1000 °C due to finalize the chemical reactions as Li doped ZnO.

**Figure 2:**
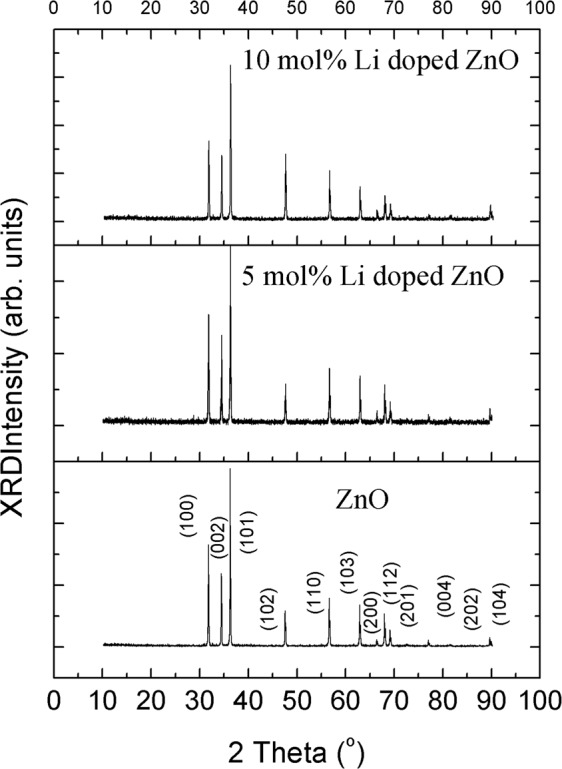
X-ray powder diffraction patterns of synthesized samples (Patterns were indexed according to Zinc Oxide (ZnO) pattern, ICDS card of PDF#00-036-1451).

According to thermal analyses, all samples with different Li doping molar ratios were annealed at 1000 ^o^C. The XRD patterns of samples with different Li doping molar ratios were shown in Fig. 2. The patterns were almost performed similar diffractions, which are accordance with Zinc Oxide (ZnO) pattern, ICDS card of PDF#00-036-1451. No additional diffraction patterns were related that the samples were produced without an impurity phase or unreacted element. In order to identify Li atom replacement in ZnO lattice, the Reitveld refinements were performed on the patterns shown in Fig. 2.

**Figure 3:**
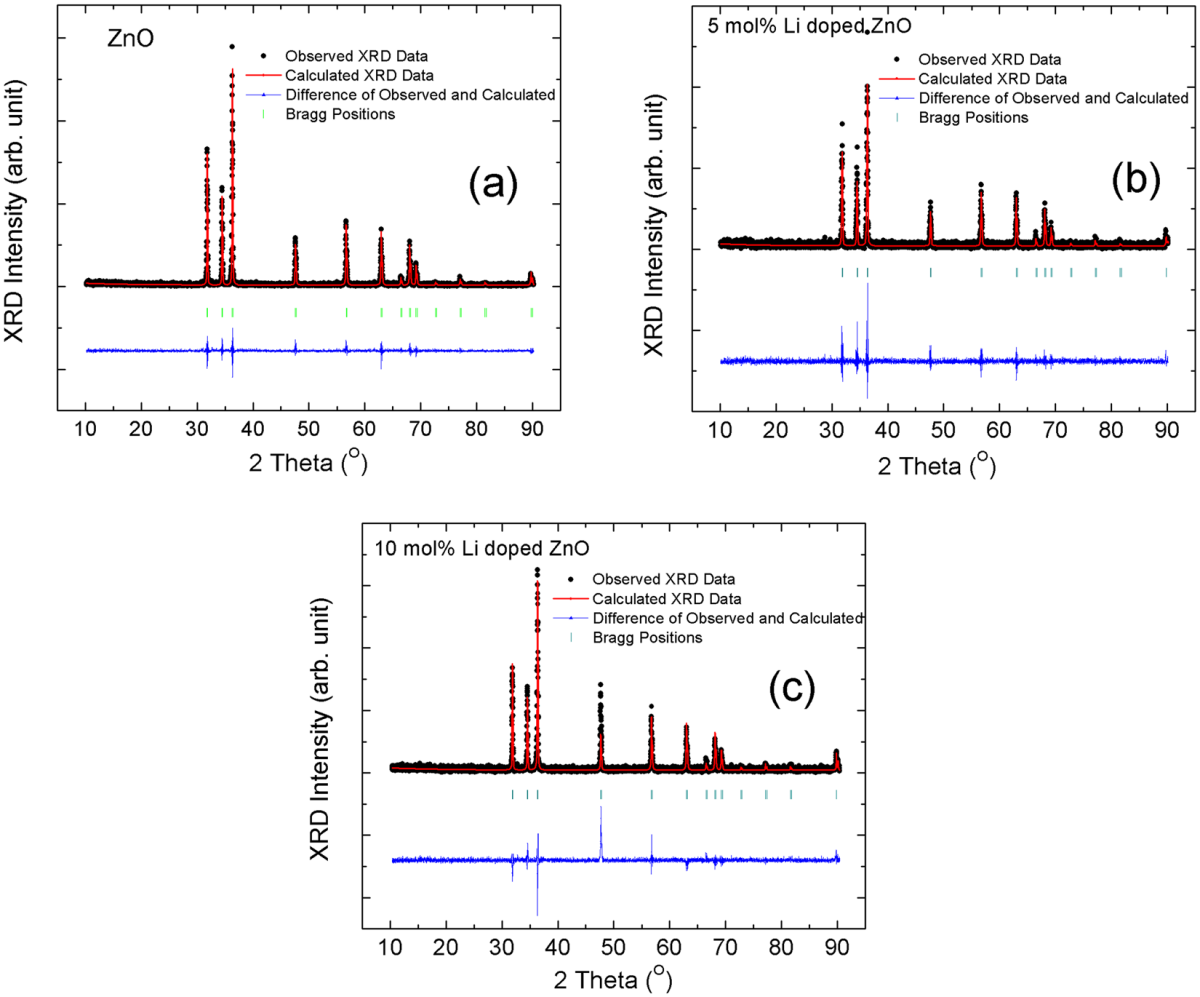
Reitveld refinements of (**a**) ZnO, (**b**) 5 mol% Li doped ZnO and (**c**) 10 mol% Li doped ZnO x-ray powder diffraction patterns.

ZnO has a hexagonal crystal structure with P6_3_mc Space group^31^. Zn^2+^ ions settle at ($\frac{1}{3}$, $\frac{2}{3}$, $0$) in the ZnO lattice^32,33^. In the calculations, we assume two different defect states, Li atoms substitute with Zn atoms and interstitial Li atoms. According to these defect states, two separate refinements were performed and the calculations were shown on Table 1. Reitveld refinements on Fig. 3 were recorded for the substitution of Li atoms with Zn atoms in the lattice. Figure 3a–c were calculated for ZnO, 5 mol% Li doped ZnO and 10 mol% Li doped ZnO, respectively.

**Table 1:** Calculated lattice parameters by employing Reitveld refinement.

*Compound*	Li location	χ^2^	R Factor	a (Å)	c (Å)
ZnO	—	1.38	5.050	3.2475	5.2016
5 mol% Li doped ZnO	Li_Zn_	1.33	11.44	3.2448	5.1976
	Li_interstitial_	1.52	10.86	3.2490	5.2041
10 mol% Li doped ZnO	Li_Zn_	1.49	12.53	3.2437	5.1258
	Li_interstitial_	1.40	11.70	3.2433	5.1953

**Figure 4:**
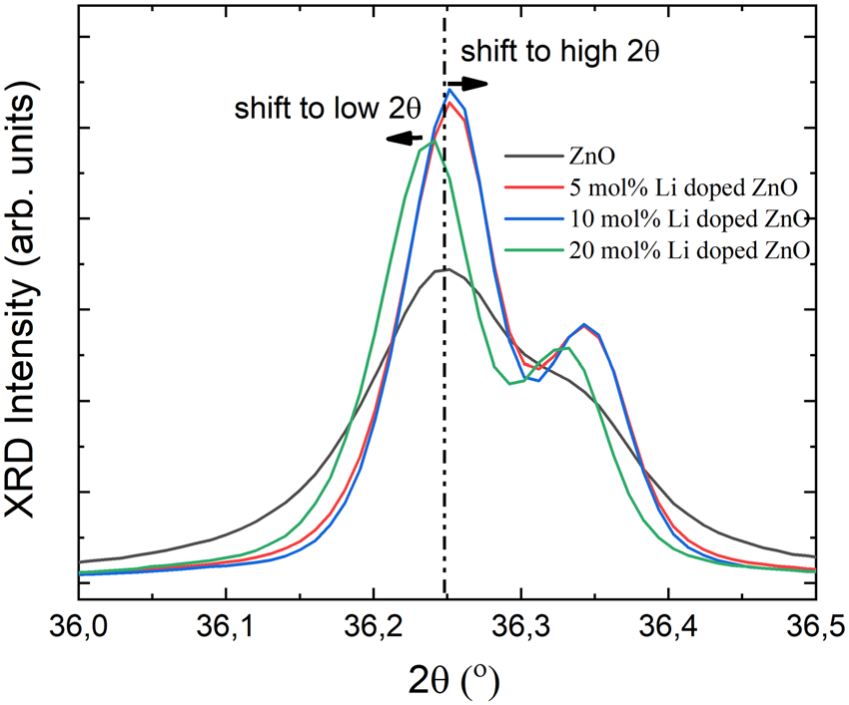
Shift in x-ray diffraction data by Li doping amount.

Li atom replacements into ZnO crystal lattice were also modified the lattice parameters as identified from a shift at diffraction peak positions^34,35^. The shift at diffraction peak positions of (100), (002) and (101) planes were illustrated on Fig. 4. As seen on Fig. 4, while the peak positions were shifted to low two theta positions for Li doping amounts lower than 3 mol%. However, high Li amount, more than 3 mol%, in ZnO lattice caused a shift at the peak positions to high 2 theta positions.

**Figure 5:**
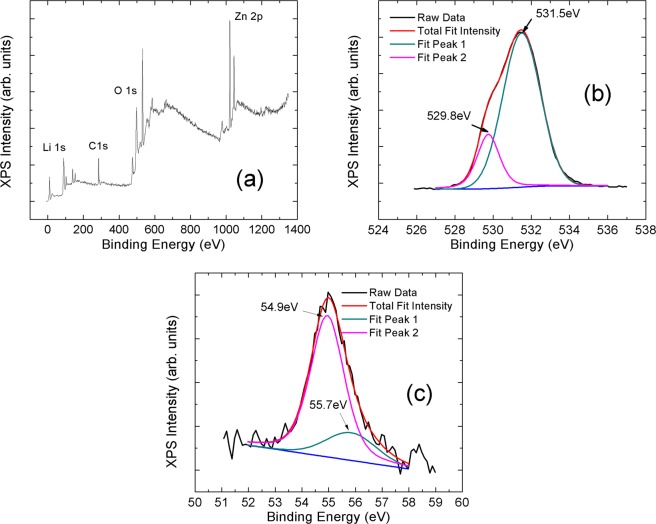
(**a**) X-ray photoelectron spectrometer measurements of 10 mol% Li doped ZnO sample, (**b**) scan for O 1 s and (**c**) L 1 s binding energy levels.

Moreover, XPS measurements were employed for the structural analyses. XPS measurements provided a comparison between expected positions of Li atoms in ZnO lattice and Reitveld refinements. C 1 s binding energy level, 284.5 eV, were used as a reference energy level. To get rid of charging dependent shift at the spectra, all XPS spectra were shifted according to C 1 s energy level. Figure 5a showed the full-scale measurement of 10 mol% Li doped ZnO sample. On the pattern, Li 1 s, O 1 s and Zn 2p electronic energy levels were indicated. Li 1 s and O 1 s electronic energy levels were measured separately as shown in Fig. 5b,c, respectively. Furthermore, Li 1 s and O 1 s energy levels were fitted into two peaks to understand the point defects and positions of Li atoms in ZnO lattice.

**Figure 6:**
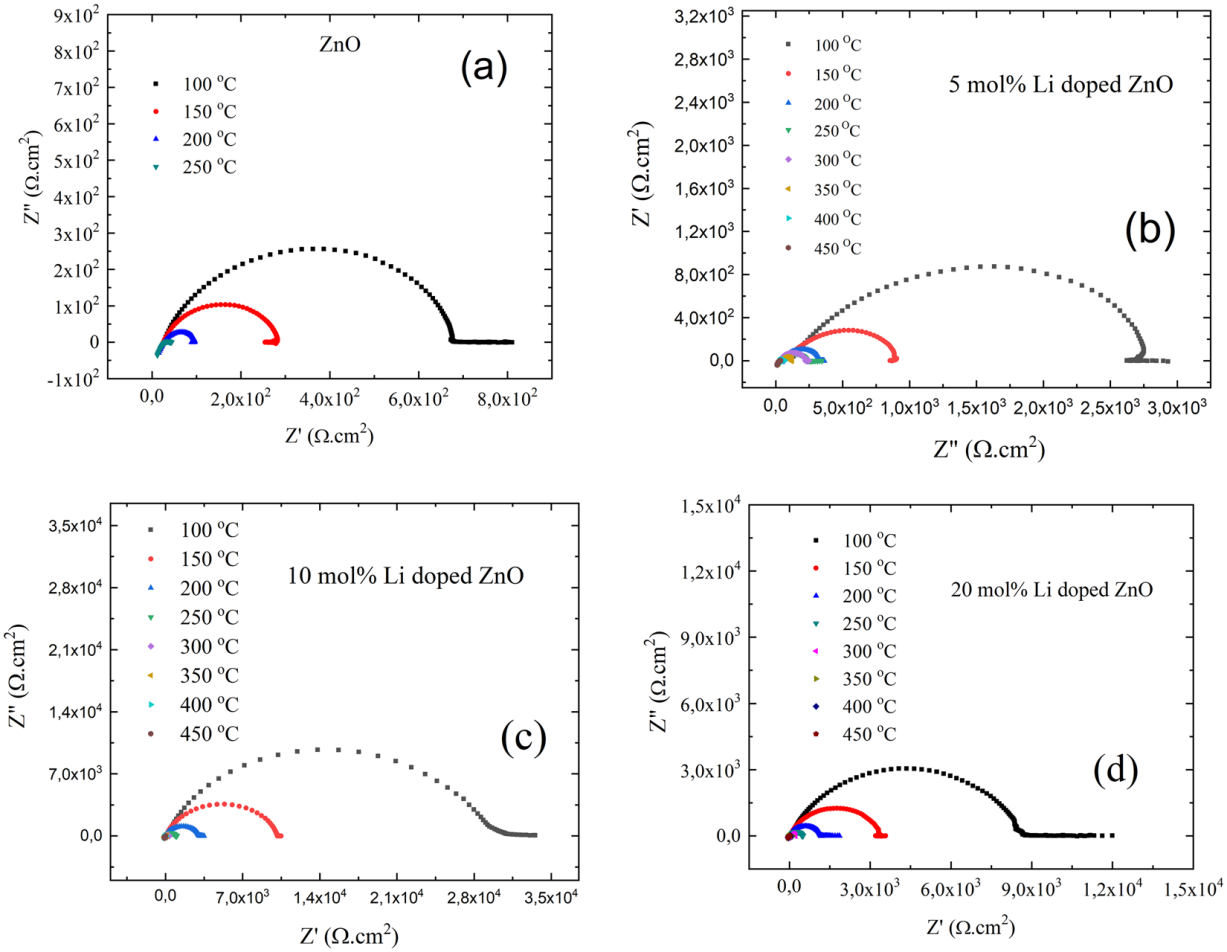
Ac impedance measurements for (**a**) ZnO, (**b**) 5 mol% Li doped ZnO, (**c**) 10 mol% Li doped ZnO and (**d**) 20 mol% Li doped ZnO pellets.

**Figure 7:**
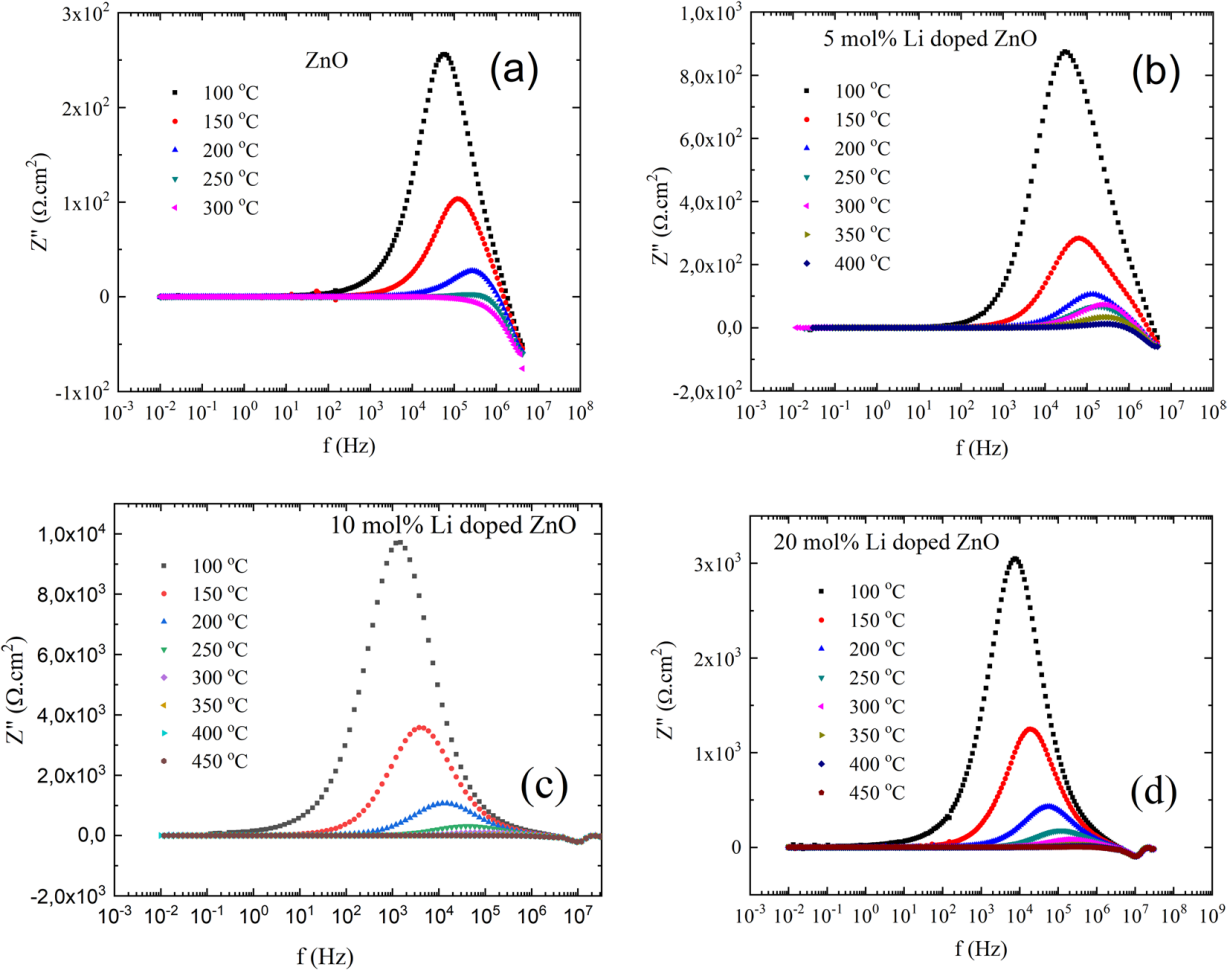
Frequency versus imaginer resistivity change of (**a**) pure, (**b**) 5 mol% Li, (**c**) 10 mol% Li and (**d**) 20 mol% Li doped ZnO pellets.

After completing structural analyses and replacement of Li atom dependent point defects in ZnO lattice, ionic transportation properties of particles were analyzed performing ac impedance measurements. The measurements were shown in Fig. 6a–d. The conductivity of ionic transportation of each pellet was measured in temperature range of 100 and 500 ^o^C. In order to specify the change of relaxation time by Li amount in ZnO lattice, frequency versus imaginer resistivity were also demonstrated in Fig. 7a–d.

## Discussion

Li doped ZnO particles were produced employing sol-gel technique. Firstly, XRD patters were determined to understand Li atoms replacement in ZnO lattice. On XRD patterns, no peak position was observed related to Li atoms or Li compounds which showed that Li atoms were replaced inside of ZnO lattice. In order to understand Li atom positions in lattice, Reitveld refinements were performed on the patterns as shown on the Table 1. The Reitveld refinements were performed according to the two assumptions which were assigned to Li atoms instead of Zn atoms and interstitial Li atoms in ZnO lattice. The hexagonal wurtzite structure of ZnO were not changed by increase in Li amount in lattice. As reported in the literature, if Li atoms prefer to be in Zn positions the lattice parameters should be decrease due to smaller ionic radius of Li^1+^ ions (0.060 nm) than Zn^2+^ ions (0.074 nm)^36,37^. In contrast the lattice parameters of ZnO should be enhanced in case of replacement of Li^1+^ ions as the interstitial defect states in ZnO lattice. As reported on Table 1, the Reitveld refinements for both conditions were fit very well. In case of Li^1+^ ions replacement in Zn^2+^ ions a small decrease in the lattice parameters, a and c, values as expected. On the other hand, no change in lattice parameters were observed for the Li^1+^ ions as the interstitial defects. According to the change in lattice parameters, Reitveld refinements indicated that Li^1+^ ions substituted with Zn^2+^ ions. In the literature, it was also mentioned that Li substituted Zn atoms at moderate concentrations doping amount, even if Li^1+^ ions were known high diffusion ability due to having low ionic radius than Zn^2+^ ions^21^.

In order to prove and understand the calculated values by Reitveld refinements, the XPS patterns were taken. On the XPS patterns Li 1 s and O 1 s binding energy levels were analyzed. The both bind energy levels, Li 1 s and O 1 s, were fit into two peak positions and the maximum values of each fit were demonstrated on Table 2. The observed peak at 55.7 eV related to Li^1+^ ions replaced to Zn^2+^ ions and bonded with O atoms as mentioned in references^23,37^. On the other hand, the peak at 54.9 eV may related to Hydroxides (Li-OH bonds on the surface) as mentioned in reference^38^. In our study no binding energy positions due to metallic Li cluster (at 52.3 eV^23,37^) and interstitial Li atoms (at 52.9 eV^23,37^) were measured in the XPS spectra.

**Table 2:** Binding energy levels of O 1 s and Li 1 s energy levels.

	O 1 s Binding Energy (eV)		Li 1 s Binding Energy (eV)	
XPS Binding Energy (experimental data)	529.8	531.5	54.9	55.7
XPS Binding Energy (in literature)	In range of 529.5 eV and 530.5 eV (the O^2-^ ions of the crystalline network^38^).	In range of 531 eV and 532 eV (oxygen ions with lower electron density than the O^2-^ ions such as O^-^ species^38^).	54.7 eV (Li-OH bonds on the surface)^38^.	55.7 eV (Li-O bonds in Zn site)^37^

In addition, the Li^1+^ ions replacement to Zn^2+^ ions were associated with O1s oxidized state. The O1s energy level was also fit into two peak positions, 529.8 eV and 531.5 eV, due to the O^2−^ ions bonded with Zn^2+^ ion, and the oxygen ions covered with low valance states such as O^1−^ ions, respectively^38^. The high binding energy level of O 1 s indicated O^1-^ ions bonded with Li^1+^ ions substituted Zn^2+^ ions.

**Figure 8:**
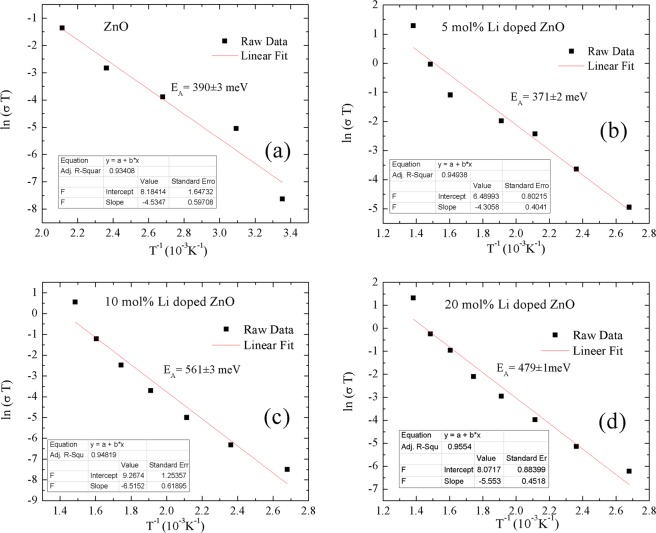
Arrhenius fits for ionic activation energy calculations of (**a**) ZnO, (**b**) 5 mol% Li doped ZnO, (**c**) 10 mol% Li doped ZnO and (**d**) 20 mol% Li doped ZnO.

After confirming the Li atom positions in ZnO lattice, Li atom amount depending ionic transportation performances of ZnO semiconductors were measured by ac impedance spectrometer. Activation energy of ionic transportation, ionic conductivity and relaxation mechanisms of ionic transportation by Li atom amount in ZnO lattice were calculated employing Nyquist plots and imaginer resistivity versus frequency curves. The frequency dependent ionic conductivity of each sample was analyzed at the constant temperature values. In addition, electronic conductivity response was identified the maximum temperature values for measurable ionic conductivities. The temperature range for ionic conductivity measurements were determined at fixed frequency (0.01 Hz) and under 100 mV voltage. The ionic conductivity measurements were performed for the high dc electrical resistivity values which was mostly out of measurement limits of the current system. And right after maximum temperature electrolyte became electrical conductor, therefore ionic conductivity was not able to be recorded. Moreover, Li doping amount was also tuned the ionic conductivity response by temperature. While ZnO pellets gave response in temperature range of 25 ^o^C and 250 ^o^C, Li doped ZnO pellets ionic conductivity responses reached in range of 100 ^o^C and 450 ^o^C. ZnO and Li doped ZnO behaved as electronically conductive above the 250 ^o^C and 450 ^o^C, respectively, at which temperature ionic conduction performance were not useful. As seen on Fig. 8, the activation energy of ionic transportation (E_A_) were obtained employing Arrhenius plots shown in Eq. (1). σ, T, σ_o_, k_B_ and E_A_ symbols were assigned for the ionic conductivity, temperature, pre-exponential constant, Boltzmann constant (-8.617×10^-5^eV.K^-1^) and the activation energy of ionic transportation, respectively. The ionic conductivity values were obtained from Nyquist plots.

1 $$\sigma T=({\sigma }_{0})exp(\frac{-{E}_{A}}{{k}_{B}T})$$

E_A_ values of ZnO, 5 mol% Li doped ZnO, 10 mol% Li doped ZnO and 20 mol% Li doped ZnO were calculated as 390 ± 3 meV, 371 ± 1 meV, 561 ± 2 meV and 479 ± 3 meV, respectively (shown in Table 3). A decrease in E_A_ value of ZnO was observed by 5 mol% Li doping. Similarly, increase in Li amount to 10 mol% was also increase the E_A_ values of ZnO. However, the E_A_ value of 20 mol% Li doping was lower than 10 mol% Li doped ZnO, but still higher than E_A_ value of undoped ZnO. Generally, the E_A_ values associated with ionic conductivity values and relaxation mechanisms of ionic conductivity. Therefore, the relaxation time changes by Li doping amount was evaluated by Eq. (2). In Eq. (2), the relaxation times of ionic carriers and the maximum peak frequency of Z″(f) curve were symbolized by τ_c_ and f_max_, respectively.

2 $$2{{\rm{\pi }}{\rm{f}}}_{{\rm{\max }}}{\tau }_{{\rm{c}}}=1$$

**Table 3:** Activation energy, conductivity and relaxation time values of ionic transportation for Li doped ZnO samples.

Compound	The activation energy for ionic conduction - E_A_ (meV)	The lowest relaxation time for ionic conductivity (µs)	The highest ionic conductivity (µS/cm^-1^)
*ZnO*	390 ± 6	0.28 ± 0.01 (250^o^C)	3436 ± 4 (250^o^C)
*5 mol% Li doped ZnO*	371 ± 4	0.39 ± 0.01 (400^o^C)	4980 ± 1 (400^o^C)
*10 mol% Li doped ZnO*	561 ± 6	0.54 ± 0.01 (400^o^C)	2570 ± 7 (400^o^C)
*20 mol% Li doped ZnO*	479 ± 5	0.34 ± 0.01 (450^o^C)	5154 ± 6 (450^o^C)

**Figure 9:**
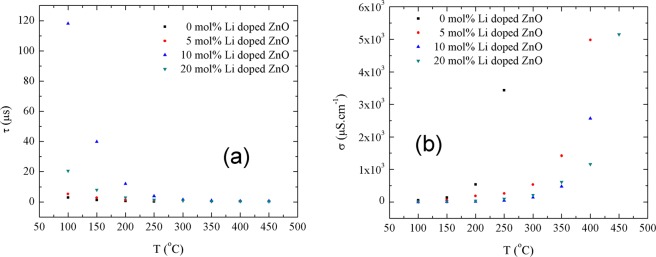
(**a**) Relaxation time and (**b**) conductivity of ionic conductivity change by temperature.

The calculated values were plotted by temperatures on Fig. 9. Obtained values at maximum temperature were demonstrated on Table 3. As seen in Fig. 9a, the highest relaxation time at 100 ^o^C were found for 10 mol% Li doped ZnO, 118.2 ± 0.1 µs. Furthermore, increase in temperature lowered the relaxation time values down to 0.28 ± 0.02 µs (250 ^o^C), 0.39 ± 0.02 µs (400 ^o^C), 0.53 ± 0.01 µs (400 ^o^C) and 0.34 ± 0.02 µs (450 ^o^C), respectively, for ZnO, 5 mol% Li doped ZnO, 10 mol% Li doped ZnO and 20 mol% Li doped ZnO. The ionic relaxation time values for Li doped ZnO were at acceptable levels to be used as electrolyte materials. In addition, the conductivity values of Li doped ZnO samples were shown in Fig. 9b. While the ionic conduction for ZnO was observed in temperature range of 25 ^o^C and 250 ^o^C, Li doped ZnO samples were ionic conductive in temperature range of 100 ^o^C and 450 ^o^C with higher conductivity values than ZnO. The highest conductions, 0.0050 ± 0.0001 S/cm, at 400 ^o^C was measured for 5 mol% Li doped ZnO and for higher temperature values than 400 ^o^C, 5 mol% Li doped ZnO became electrically conductive. On the other hand, Nyquist plots for 20 mol% Li doped ZnO were measured in temperature range of 100 ^o^C and 450 ^o^C and at 500 ^o^C the electrical conductivity for 20 mol% Li doped ZnO sample was observed. The highest conductivity for 20 mol% Li doped ZnO samples was measured as 0.0052 ± 0.0001 S/cm at 450 ^o^C.

## Conclusion

Our study demonstrated direct insights of on how to enhance the usefulness of ZnO semiconductors as electrolyte materials by doping with Li atoms. Li doped ZnO particles were synthesized employing sol-gel technique. Reitveld refinements were performed to understand the Li replacement. It was calculated that Li atoms preferred to replace instead of Zn atoms in ZnO lattice. The replacement of Li atoms in ZnO lattice were also modified the ionic transportation ability of ZnO. According to the calculated conductivity and relaxation time values, Li atoms amount enhanced the activation energies of ionic transportation, therefore, ZnO semiconductor became useful electrolyte materials by Li amount in lattice with low ionic relaxation times, 0.34 ± 0.02 µs at 450 ^o^C (for 20 mol% Li doped ZnO), and high conductivity values, 0.0052 ± 0.0001 S/cm at 450 ^o^C (for 20 mol% Li doped ZnO).

## References

[1] Yamazoe N, Sakai G, Shimanoe K (2003). Oxide semiconductor gas sensors. Catalysis Surveys from Asia.

[2] Fine GF, Cavanagh LM, Afonja A, Binions R (2010). Metal Oxide Semi-Conductor Gas Sensors in Environmental Monitoring. Sensors.

[3] Choi KJ, Jang HWJ (2010). One-Dimensional Oxide Nanostructures as Gas-Sensing Materials: Review and Issues. Sensors.

[4] Ojha S, Das AS, Roy M, Bhattacharya S (2018). Temperature and frequency response of conductivity in Ag2S doped chalcogenide glassy semiconductor. Physica B: Condensed Matter.

[5] Das AS (2018). Ac conductivity of transition metal oxide doped glassy nanocomposite systems: temperature and frequency dependency. Mater. Res. Express.

[6] Das AS, Roy M, Roy D, Bhattacharya S, Nambissan PMG (2018). Identification of defects in the transition metal oxide-doped glass nanocomposite xV2O5–(1-x)(0.05MoO3–0.95ZnO) using positron annihilation spectroscopy and other techniques. Journal of Non-Crystalline Solids.

[7] Fan L, Ma Y, Wang X, Singh M, Zhu B (2014). Understanding the electrochemical mechanism of the core–shell ceria–LiZnO nanocomposite in a low temperature solid oxide fuel cell. J Mater Chem A.

[8] Lutkenhaus JL, Hammond PT (2007). Electrochemically enabled polyelectrolyte multilayer devices: from fuel cells to sensors. Soft Matter.

[9] Shawuti S, Can MM, Gülgün MA, Fırat T (2014). Grain Size Dependent Comparison of ZnO and ZnGa2O4 Semiconductors by Impedance Spectrometry. Electrochimica Acta.

[10] Shawuti S, Gülgün MA (2016). Solid oxide carbonate composite fuel cells: Size effect on percolation. International Journal of Hydrogen Energy.

[11] Qiao Z (2018). Electrochemical and electrical properties of doped CeO2-ZnO composite for low-temperature solid oxide fuel cell applications. Journal of Power Sources.

[12] Xia C (2018). Study on Zinc Oxide-Based Electrolytes in Low-Temperature Solid Oxide Fuel Cells. Materials.

[13] Xia C (2019). Shaping triple-conducting semiconductor BaCo0.4Fe0.4Zr0.1Y0.1O3-δ into an electrolyte for low-temperature solid oxide fuel cells. Nature Communications.

[14] Kreuer KD (2001). Proton conducting alkaline earth zirconates and titanates for high drain electrochemical applications. Solid State Ionics.

[15] Shawuti S, Can MM, Gülgün MA, Kaneko S, Endo T (2018). Influence of grain boundary interface on ionic conduction of (Zn1-x,Cox)O. Composites Part B: Engineering.

[16] Li Y, Rui Z, Xia C, Anderson M, Lin YS (2009). Performance of ionic-conducting ceramic/carbonate composite materialas solid oxide fuel cell electrolyte and CO2 permeation membrane. Catalysis Today.

[17] Thangadurai V, Weppner W (2006). Recent progress in solid oxide and lithium ion conducting electrolytes research. Ionics.

[18] Wu J (2012). A novel core–shell nanocomposite electrolyte for low temperature fuel cells. Journal of Power Sources.

[19] Accardo G, Ferone C, Cioffi R (2016). Influence of Lithium on the Sintering Behavior and Electrical Properties of Ce0.8Gd0.2O1.9 for Intermediate Temperature Solid Oxide Fuel Cells. Energy Technol.

[20] Xia C (2018). Semiconductor electrolyte for low-operating-temperature solid oxide fuel cell: Li-doped ZnO. International Journal of Hydrogen Energy.

[21] Yi JB (2010). Ferromagnetism in Dilute Magnetic Semiconductors through Defect Engineering: Li-Doped ZnO. Phys Rev Lett.

[22] Zeng Y-J (2005). Realization of p-type ZnO films via monodoping of Li acceptor. Journal of Crystal Growth.

[23] Lu JG (2006). Control of - and -type conductivities in Lidoped ZnO thin films. Applied Physics Letters.

[24] Raza Rizwan, Zhu Bin, Rafique Asia, Naqvi MuhammadRaza, Lund Peter (2020). Functional ceria-based nanocomposites for advanced low-temperature (300–600 °C) solid oxide fuel cell: A comprehensive review. Materials Today. Energy.

[25] Acharya A, Bhattacharya K, Ghosh CK, Bhattacharya S, Microstructures and Charge Carrier Transport of Some Li2O doped Glassy Ceramics, Materials Letters, (2020) in press.

[26] Bhattacharya S, Acharya A, Biswas D, Das AS, Singh LS (2018). Conductivity spectra of lithium ion conducting glassy ceramics. Physica B: Condensed Matter.

[27] Bhattacharya S, Acharya A, Das AS, Bhattacharya K, Ghosh CK (2019). Lithium ion conductivity in Li2OeP2O5-ZnO glass-ceramics. Journal of Alloys and Compounds.

[28] Rodriguez-Carvajal J (1993). Recent advances in magnetic structure determination by neutron powder diffraction. Physica B.

[29] Zak AK, Aziz NSA, Hashim AM, Kordi F (2016). XPS and UV–vis studies of Ga-doped zinc oxide nanoparticles synthesized by gelatin-based sol-gel approach. Ceramics International.

[30] Shi L, Tan YS, Tsubaki N (2012). A Solid-State Combustion Method towards Metallic Cu–ZnO Catalyst without Further Reduction and its Application to Low-Temperature Methanol Synthesis. Chem Cat Chem.

[31] Jayakumar OD, Gopalakrishnan IK, Kulshreshtha SK (2006). Surfactant-Assisted Synthesis of Co- and Li-Doped ZnO Nanocrystalline Samples Showing Room-Temperature Ferromagnetism. Adv Mater.

[32] Singhal RK (2010). Electronic and magnetic properties of Co-doped ZnO diluted magnetic semiconductor. Journal of Alloys and Compounds.

[33] Kumar V, Kumari S, Kumar P, Kar M, Kumar L (2015). Structural analysis by rietveld method and its correlation with optical properties of nanocrystalline zinc oxide. Adv Mater Lett.

[34] Wang B, Tang L, Qi J, Du H, Zhang Z (2010). Synthesis and characteristics of Li-doped ZnO powders for p-type ZnO. Journal of Alloys and Compounds.

[35] Hjiri M, Aida MS, Lemine OM, El Mir L (2019). Study of defects in Li-doped ZnO thin films. Materials Science in Semiconductor Processing.

[36] Srinivasan G, Kumar RTR, Kumar J (2007). Li doped and undoped ZnO nanocrystalline thin films: a comparative study of structural and optical properties. J Sol-Gel Sci Technol.

[37] Awan SU, Hasanain SK, Bertino MF, Jaffari GH (2012). Ferromagnetism in Li doped ZnO nanoparticles: The role of interstitial Li. J Appl Phys.

[38] Dupin JC, Gonbeau D, Vinatier P, Levasseur A (2000). Systematic XPS studies of metal oxides, hydroxides and peroxides. Phys Chem Chem Phys.

